# Phosphoproteome Reveals the Role of Baicalin in Alleviating rPVL-Induced Cell Cycle Arrest in BMECs

**DOI:** 10.3390/microorganisms13071673

**Published:** 2025-07-16

**Authors:** Ling Hou, Jun Li, Juqing Wang, Qin You, Dongtao Zhang, Xuezhang Zhou

**Affiliations:** 1 Key Laboratory of the Ministry of Education for the Conservation and Utilization of Special Biological Resources of Western China, Ningxia University, Yinchuan 750021, China; hlbelief@163.com (L.H.); muzi3202@163.com (J.L.);; 2 Institute of Basic Medical Science, Ningxia Medical University, Yinchuan 750004, China; 3 Ningxia Research Center for Development and Utilization of Chinese Medicinal Materials, Ningxia Polytechnic, Yinchuan 750021, China

**Keywords:** recombinant Panton–Valentine leukocidin (rPVL), baicalin, cell cycle arrest, breast tissue injury, 4D-DIA phosphorylated proteomics

## Abstract

Panton–Valentine leukocidin (PVL) is a pore-forming toxin secreted by *Staphylococcus aureus* (*S. aureus*) and a significant virulence factor that plays a crucial role in the pathogenesis of dairy mastitis. Previous studies by our research group demonstrated that baicalin inhibits the apoptosis and hyperphosphorylation of cytoskeletal proteins induced by recombinant Panton–Valentine leukocidin (rPVL) in bovine mammary epithelial cells (BMECs). However, the effects of baicalin on the proliferation of BMECs and the underlying mechanism remain unclear. Consequently, this study aimed to explore this underlying mechanism through an LC-MS/MS analysis performed in 4D data-independent acquisition (DIA) mode. Quantitative analysis identified 757 differentially expressed phosphoproteins, among which phosphorylation levels of proteins involved in BMEC proliferation and cell cycle regulation exhibited significant alterations (*p* < 0.05). rPVL inhibited BMEC proliferation in a dose-dependent manner and induced G0/G1 phase arrest and dephosphorylation of the cell-cycle-related proteins BCLAF1^S285^, CDK7^T170^, NF2^S518^, and PKM2S37. Preintervention with baicalin significantly upregulated the expression and phosphorylation of these proteins and alleviated the G0/G1 phase arrest induced by rPVL in BMECs *in vitro*. The establishment of the mitotic state in BMECs due to the effect of baicalin appears to be closely related to the regulation of the phosphorylation of CDK7, PKM2, BCLAF1, and NF2. Moreover, *in vivo* analysis revealed that *S. aureus* ATCC49775 and rPVL induced dramatic structural destruction and pathological impairment of mammary gland tissues in mice and that these histopathological changes were ameliorated after baicalin intervention. Quantitative immunohistochemical analysis revealed that baicalin mitigated the rPVL-induced dephosphorylation of the aforementioned cell-cycle-related proteins and increased their phosphorylation. Both *in vitro* and *in vivo* experimental evidence demonstrated that baicalin effectively reversed rPVL-induced G0/G1 phase arrest in BMECs (*p* < 0.01) by significantly increasing the phosphorylation levels of cell cycle regulatory proteins (*p* < 0.05). Additionally, baicalin alleviates pathological damage to mammary gland tissues in mouse models. These data suggest that baicalin possesses antibacterial and antitoxin effects, indicating that it is an effective preventive agent against bovine mastitis.

## 1. Introduction

*Staphylococcus aureus* is an important veterinary and zoonotic pathogen and the leading cause of clinical mastitis, which causes great economic losses and leads to reduced milk quality and production. Mastitis may threaten human health through the spread of methicillin-resistant *S. aureus* (MRSA), which has become a significant concern for both animal health and public health [1,2,3]. A major contributor to the success of *S. aureus* as a pathogen and its ability to infect is related to the plethora of virulence factors in this species [4].

Pore-forming toxins (PFTs) constitute a crucial virulence strategy for bacterial pathogens, such as MRSA [5,6,7]. PFTs disrupt host cell membranes to internalize or transmit other bacterial or virulence factors and thereby establish infection. Evidence also suggests that the recognition of PFTs in host cells activates multiple signal transduction pathways [8,9,10]. Panton–Valentine leukocidin (PVL), which is secreted by *S. aureus* and causes mastitis in ruminants, is a two-component PFT that consists of the LukS-PV and LukF-PV subunits [11]. PVL mediates toxin binding and cytotoxicity by targeting the C5a receptor on host cells [12]. Notably, the expression of this receptor on bovine mammary epithelial cells has been experimentally confirmed [13]. Emerging evidence from several laboratories shows that the *pvl* gene and MRSA with the *pvl* gene are being increasingly detected in raw milk from dairy cows with mastitis [14,15,16,17]. PVL reportedly induces apoptosis and inhibits proliferation in cells from various sources [18,19,20,21]. These data indicate that PVL is a significant cause of injury to bovine mammary epithelial cells (BMECs). Previous studies by our research group revealed that the rates of apoptosis and necrosis induced by the standard PVL-producing strain of *S. aureus* (ATCC 49775) and the complemented mutant C-Δpvl 49775 were significantly greater than those induced by the PVL knockout mutant Δpvl 49775 in BMECs, demonstrating that apoptosis and necrosis in BMECs are associated with PVL. Additionally, recombinant PVL (rPVL) also induced apoptosis in BMECs *in vitro*, suggesting that recombinant PVL has a virulence effect similar to that of wild-type PVL [15,22]. However, the effects of rPVL on the proliferation of BMECs and the underlying mechanism remain unclear.

Baicalin, the major bioactive flavonoid in *Scutellaria baicalensis* Georgi (SBG) roots, is incorporated into animal feed in China [23]. Baicalin has been shown to have many beneficial effects, including anti-inflammatory, antibacterial, antioxidant, antiviral, and antitumor effects [24,25]. Previous studies by our research group have shown that baicalin can significantly reverse apoptosis induced by both *S. aureus* ATCC49775-producing PVL and recombinant PVL (rPVL) in BMECs *in vitro* [15,22,26]. However, owing to the complexity of the biological processes mediated by baicalin, the impact of baicalin on PVL-induced cell cycle arrest remains unexplored.

Post-translational modifications influence the function and fate of proteins and cells in various ways [27]. Changes in signal networks mediated by protein phosphorylation and dephosphorylation are closely related to the occurrence and development of many diseases [15,28]. Four-dimensional (4D) data-independent acquisition (DIA)-based phosphoproteomics involves large-scale analysis of protein phosphorylation sites and has emerged as a powerful tool for defining signal network regulation or dysregulation under normal and pathological conditions [29]. This study employed 4D-DIA phosphoproteomics to investigate the mechanism through which baicalin alleviates rPVL-induced cell cycle arrest.

## 2. Materials and Methods

### 2.1. Bacterial Strains and rPVL Production and Purification

*S. aureus* ATCC49775-producing PVL was purchased from the American Type Culture Collection (ATCC). The bacteria were cultured overnight in tryptic soy agar broth (Hai Bo Co. Ltd., Qingdao, China) under shaking conditions (120 rpm) at 37 °C. For infection, *S. aureus* was cultured until the mid-log phase, collected through centrifugation, washed with sterile phosphate-buffered saline (PBS), and diluted to the required concentration (MOI = 30). *E. coli* BL21 and the expression vector pET28a for the expression and purification of rPVL were purchased from Sangon Biotech (Shanghai, China). The gene sequences of the LukF-PV protein and LukS-PV protein of PV-leukocidin were inserted into the pET28a expression vector, and the vector was transformed into *E. coli* receptor cells, which were heat-excited at 42 °C. The cells were streaked onto agar plates containing 30 µg/mL kanamycin, cultured at 37 °C, and induced to express. The recombinant LukF-PV protein and LukS-PV protein were subjected to affinity purification, and the purity was confirmed via SDS-PAGE and Western blotting (purity > 90%). Detailed sequence information, experimental methods, and protein expression and purification processes for LukF-PV and LukS-PV are provided in Supplementary Materials S1 and S2.

### 2.2. Cell Culture and Sample Treatment

The bovine mammary epithelial cell line MAC-T was obtained from Shandong Agricultural University, Tai’an, China. The MAC-T-cell line was cultured in Dulbecco’s modified Eagle’s medium (DMEM) (Gibco, Waltham, MA, USA) supplemented with 10% fetal bovine serum (*v*/*v*) (Gibco, Waltham, MA, USA) along with 100 µg/mL gentamicin and 100 µg/mL penicillin–streptomycin (HyClone, Logan, UT, USA) at 37 °C in a 5% CO_2_ atmosphere until 80–90% confluency was reached. The two treatment groups included BMECs treated with rPVL (rPVL) for 3 h and BMECs incubated with rPVL after pretreatment with 5 μg/mL baicalin for 3 h (Bai+rPVL). The final concentration of rPVL in the treatment groups was 100 ng/mL. The control group (Con) included cells in fresh DMEM without rPVL or baicalin. Each group included three biological replicates.

### 2.3. SDT Digestion of Whole-Cell Lysates and LC-MS/MS Analysis in 4D Data-Independent Acquisition (DIA) Mode

SDT buffer (4% SDS, 100 mM Tris-HCl, pH 7.6) was added to the samples (Con, rPVL, and Bai+rPVL groups), and the lysate was sonicated and then boiled for 15 min. After centrifugation at 14,000× *g* for 15 min, the supernatant was quantified via a BCA protein assay kit (P0012, Beyotime, Shanghai, China). Protein (20 µg) from each sample was mixed with 6X loading buffer and boiled for 5 min. Afterward, the proteins were separated on a 12% SDS-PAGE gel. A total of 600 µg of protein from each sample was subjected to FASP digestion. The labeled peptides were combined and desalted via a C18 column. The samples were analyzed on a nanoElute (Bruker, Bremen, Germany) coupled to a tims TOF Pro (Bruker, Bremen, Germany), which was operated in PASEF mode. The raw DIA data were processed and analyzed using Spectronaut (Biognosys AG, Schlieren, Switzerland) with default settings.

### 2.4. Cell Cycle Analysis

BMECs were seeded at a density of 5 × 10^5^ in the wells of 6-well plates, followed by overnight incubation at 37 °C. The two treatment groups included BMECs treated with various concentrations (100 ng/mL, 1 µg/mL, 5 µg/mL, or 10 µg/mL) of rPVL for 12 h and BMECs incubated with various concentrations of rPVL as described above after pretreatment with 5 μg/mL baicalin for 12 h (Bai+rPVL). The cells were collected through centrifugation at 1500 rpm for 5 min, washed with ice-cold PBS, and fixed with 70% ice-cold ethanol at 4 °C for 24 h. Cell cycle analysis was performed via a cell cycle kit according to the manufacturer’s instructions (Beyotime, Shanghai, China), and the results were detected using a BD AccuriTM C6 Plus (BD Biosciences, Mountain View, CA, USA). The data were analyzed with FlowJo software10.8.1.

### 2.5. Western Blot Analysis

To determine protein levels, total protein was extracted from BMECs using a whole-cell lysis assay (Key GEN Bio TECH, Nanjing, Jiangsu, China) and quantified using a BCA protein assay kit (Key GEN Bio TECH, Nanjing, Jiangsu, China) according to the manufacturer’s instructions. After boiling for 10 min, the total protein samples (20 µg/well) were loaded onto 7.5% sodium dodecyl sulfate–polyacrylamide gel electrophoresis (SDS-PAGE) gels and transferred to polyvinylidene difluoride (PVDF) membranes (Millipore, Burlington, MA, USA) at a constant current of 300 mA for 90 min. Then, the membranes were incubated overnight with the corresponding primary antibodies at 4 °C after blocking with 5% non-fat dry milk in Tris-buffered saline (TBS)-Tween-20 (TBST) for 1 h at room temperature. After three washes with TBST, the membranes were incubated with a horseradish peroxidase (HRP)-conjugated goat anti-rabbit IgG antibody (1:10,000) for 1 h at room temperature, after which the protein bands were visualized via an enhanced chemiluminescence (ECL) reagent (Perkin Elmer, Waltham, MA, USA). NF2 (1:7000, Cat. No. 21686–1-AP), P-NF2 (Ser518, 1:7000, Cat. No. 28851–1-AP), CDK7 (1:2800, Cat. No. 27027–1-AP), and PKM (1:2800, Cat. No. 10078–2-AP) antibodies were purchased from Proteintech (Rosemont, IL, USA). P-CDK7 (Thr170, 1:2000, Cat. No. bs-10997R) antibody was purchased from Bioss (Beijing, China). P-PKM2 (Ser37, 1:1400, Cat. No. AF7231), BCLAF1 (1:1000, Cat. No. DF3828), and P-BCLAF1 (Ser531, 1:2000, Cat. No. AF8245) antibodies were purchased from Affinity (Liyang, Jiangsu, China).

### 2.6. Quantitative Immunofluorescence (IF) Analysis

BMECs were treated according to the experimental design (as described in Section 2.2). The medium was removed, and the cells were washed three times with PBS every 5 min. Then, the cells were fixed with 4% paraformaldehyde (SEVEN BIOTECH, Beijing, China) for 10 min, and the fixing solution was discarded. After being washed with PBS, the cells were permeabilized with 0.2% Triton X-100 (Cat#T8200, Solarbio, Beijing, China) for 10 min and blocked for 1 h with 5% BSA (Cat#A8020, Solarbio) at 37 °C. The cells were then incubated with primary antibodies (BCLAF1^S285^, CDK7^T170^, NF2^S518^, and PKM2^S37^ antibodies at a dilution of 1:500 in antibody diluent) overnight at 4 °C. After the cells were washed three times with PBS every 5 min to remove the unbound primary antibodies, incubation with the secondary antibodies was performed for 1 h at 37 °C in the dark with CoraLite488-conjugated goat anti-rabbit IgG (H+L) and CoraLite594-conjugated goat anti-rabbit IgG (H+L) at a dilution of 1:1000 in 1× PBS. The coverslips were mounted on a microscope slide using mounting medium after counterstaining with 5 mg/mL 2-(4-amidinophenyl)–6-indolecarbamidine (DAPI; Beyotime, China) for 5 min. The samples were analyzed via fluorescence microscopy (OMPUS IX73, Tokyo, Japan). The fluorescence intensity was quantified via ImageJ v1.8.0 software, and normalization processing was performed.

### 2.7. Mouse Experimental Groups and Drug Treatments

ICR mice (6–8 weeks old, 20–25 g in weight) were procured from Huachuang SinoPharmaTech Co. (Taizhou, Jiangsu, China) and provided ad libitum access to food and water. The mice were distributed in small cages, with each cage having three females and one male, after one week of acclimatization. Sixty lactating mice were randomly divided into 6 groups (n  =  10 per group) one week after parturition: control (blank), control (0.9% NaCl, 50 µL), ATCC49775 (1 × 10^9^ CFU per 50 µL), rPVL (0.2 mg/mL, 50 µL), Bai+ATCC49775 (100 mg/kg), and Bai+rPVL (100 mg/kg). Baicalin was dissolved in physiological saline. The mice were intraperitoneally injected with baicalin three times (at 6, 12, and 18 h) within the first 24 h before infection was induced. An *S. aureus* suspension and rPVL were injected into the fourth canal of each mouse via a microsyringe. All of the mice were sacrificed after 24 h, and their mammary gland samples were collected. All mouse experiments used in this study were approved by the Institutional Animal Care and Use Committee of Ningxia University (approval no. 23–74).

### 2.8. Hematoxylin–Eosin (H&E) Staining

Mouse mammary tissue was collected and fixed in 4% paraformaldehyde, followed by dehydration, transparency, and paraffin embedding. Afterward, the samples were sliced into 4 µm longitudinal sections and stained with hematoxylin solution for 5 min, followed by immersion in 1% acid ethanol (1% HCl in 70% ethanol) for 3 s and then rinsing with distilled water. For counterstaining, the sections were stained with eosin solution for 3 min, dehydrated with graded alcohol, and subjected to xylene until transparency; each step lasted 5 min. The mounted slides were then photographed under a SOPTOP EX30 fluorescence microscope (Ningbo, China).

### 2.9. Quantitative Immunohistochemical Analysis

Longitudinal sections (4 μm in thickness) of the paraffin-embedded mammary gland tissues were incubated in an oven at 65 °C for 1 h, deparaffinized with xylene, and dehydrated using graded ethanol (100–70%). The appropriate antigen retrieval method was subsequently implemented. The sections were then incubated in 3% H_2_O_2_ for 10 min, blocked with 5% normal goat serum for 20 min, and incubated with primary antibodies (BCLAF1^S285^, CDK7^T170^, NF2^S518^, and PKM2^S37^ antibodies, 1:100) overnight at 4 °C. After 18–24 h, the slides were rewarmed at room temperature for 45 min, followed by incubation with the corresponding secondary antibody (horseradish peroxidase (HRP)-conjugated goat anti-rabbit IgG antibody, 1:100) at 37 °C for 20 min, 3,3′-diaminobenzidine (DAB) for 3 min, and dehydration with an ethanol gradient (70–100%). Finally, the sections were sealed with neutral adhesive. DAB staining was performed and analyzed using a Halo 101-WL-HALO-1 image analysis system.

### 2.10. Statistical Analysis

All of the experiments were performed in triplicate. All of the resulting data are expressed as the means ±standard deviations (SDs). Statistical differences between groups were determined via Student’s t-test. The data were analyzed and graphed using GraphPad Prism 8 (GraphPad Software, San Diego, CA, USA). A value of *p* < 0.05 was considered statistically significant. The raw DIA data were processed and analyzed using Spectronaut (Biognosys AG, Switzerland) with default parameters, and the retention time prediction type was set to dynamic iRT. Spectronaut can dynamically determine the ideal extraction window on the basis of iRT calibration and gradient stability. A Q value cutoff of 1% was applied at the precursor and protein levels. After the Student’s *t*-test, the differentially expressed proteins were filtered according to the following criteria: *p* < 0.05 and a fold change > 1.5. Multiple testing correction was applied using the false discovery rate (FDR) based on the Benjamini–Hochberg (BH) method.

## 3. Results

### 3.1. Differentially Expressed Phosphorylated Proteins (DEPPs)

**Figure 1:**
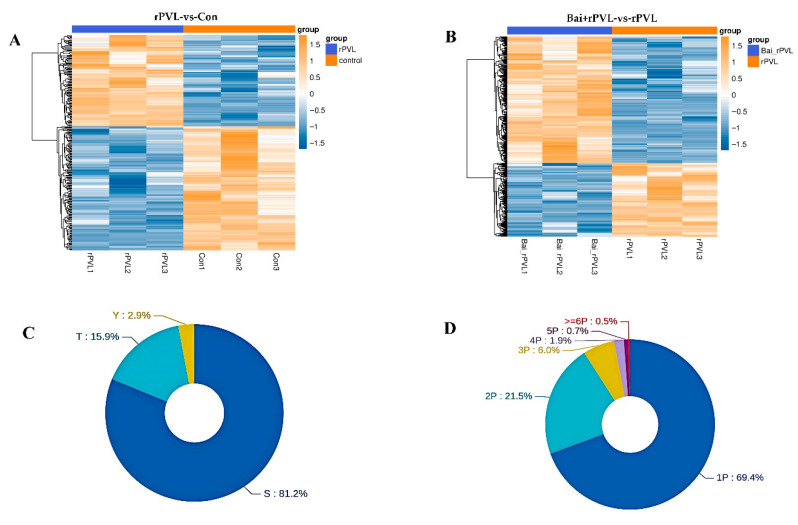
Statistical map of the phosphorylation identification results (localization probabilities > 75%). (**A**,**B**) The clustering analysis categorized data across both sample and variable dimensions, evaluating the validity of the selected target phosphorylation sites through intergroup comparisons. In this framework, each column represents an individual sample, and distinct groups are indicated using bars of varying colors. Each row corresponds to a different phosphorylation site, and the relative abundance of these sites is visually depicted through color variation, where yellow indicates high expression and blue signifies low expression. (**C**) Modified sites in the modified peptides, illustrating the distribution of the S/T/Y phosphorylated modification sites at the amino acid level. (**D**) Number of phosphorylated sites showing the quantitative distribution of the phosphorylated modification sites at the peptide level.

Strict data filtering was used in this experiment. The accuracy FDR for peptide- and protein-level identification was set to 1%. The localization likelihood of the phosphorylation sites was set to 0.75. Solid-phase metal ion affinity chromatography (IMAC) and metal oxide affinity chromatography (MOAC) were applied to enrich the phosphorylated peptides. The DIA quantitative method was used to obtain qualitative and quantitative results for the phosphorylated peptides in several samples. Before the cluster analysis, the quantitative values of the target proteins were normalized, and then the Matplotlib 3.3.3 package in Python 3.9.5 was applied to classify the samples and protein expression at the same time. Finally, the hierarchical clustering heatmap was generated. In this study, specifically, the experiments focused on examining the impact of rPVL on the phosphorylation processes occurring in BMECs and how baicalin, when administered prior to rPVL exposure, modulates these cellular responses. A total of 757 phosphorylated proteins were identified, among which 274 (116 upregulated and 158 downregulated) and 483 (305 upregulated and 178 downregulated) phosphorylated sites were differentially expressed between the two comparison groups (rPVL-vs-Con and Bai+rPVL-vs-rPVL, respectively; Figure 1A,B). Among these sites, serine (S) sites constituted 81.2%, threonine (T) sites constituted 15.9%, and tyrosine (Y) sites constituted 2.9% of the total number of sites (Figure 1C). A total of 69.4% of the peptides were phosphorylated at one site, and a small number of peptides were phosphorylated at multiple sites (Figure 1D).

### 3.2. KEGG Pathway and Gene Ontology (GO) Analysis of DEPPs

**Figure 2:**
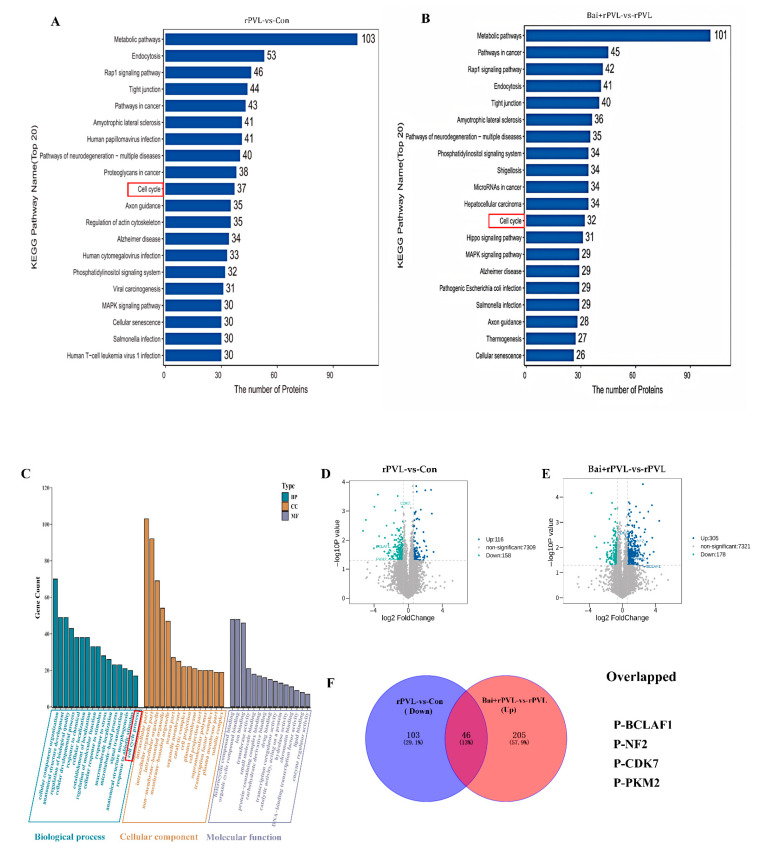
KEGG Pathway and Gene Ontology (GO) Analysis of DEPPs. (**A**,**B**) A histogram of the top 20 KEGG pathways that were significantly regulated in terms of phosphorylation in the two groups for comparison. The X-axis represents the number of proteins, and the Y-axis indicates the distinct KEGG pathways. (**C**) Bar chart for the GO annotations, depicting upregulated and downregulated genes at level 3. The GO analysis results are summarized in three categories: biological processes (blue), cellular components (yellow), and molecular functions (purple). The X-axis shows different GO terms, and the Y-axis indicates the number of genes. (**D**,**E**) Volcano plots showing the differentially expressed proteins (DEPPs) between the two groups. DEPPs are shown as blue (upregulated) and green (downregulated) dots. Non-significantly expressed proteins are shown as gray dots. The X-axis represents the log2 value (fold change), and the Y-axis represents the −log10 value (*p* value). (**F**) Venn diagrams depicting the overlap of downregulated phosphosites after rPVL-induced damage in BMECs and upregulated phosphosites after baicalin intervention. The overlapping phosphorylated proteins that regulate cell proliferation are listed below.

For the KEGG pathway annotation process for the DEPPs, KOALA (KEGG Orthology And Links Annotation, V2.3) software was used to align the KEGG GENES database with the DEPPs. The DEPP sequences were subsequently classified via the KO label, and the pathway information was obtained automatically. The KEGG annotation analysis of the DEPPs revealed 4381 DEPPs in the 304 KEGG pathways of the rPVL-vs-Con group and 4431 DEPPs in the 296 KEGG pathways of the Bai+rPVL-vs-rPVL group. The phosphosites to KEGG signaling pathways were subsequently mapped, and the top 20 KEGG pathways, according to the number of proteins to which the differential phosphosites belong, were selected. The DEPPs were significantly enriched in pathways related to the cell cycle, apoptosis, cell population proliferation, regulation of the actin cytoskeleton, the rap1 signaling pathway, and the MAPK signaling pathway. In the phosphoproteome, rPVL was verified to downregulate the cell cycle pathway, whereas baicalin preintervention upregulated the cell cycle pathway (Figure 2A,B). The focus was on the pathways associated with the cell cycle. The results of the KEGG analysis were consistent with those of the GO enrichment analysis.

The GO enrichment analysis revealed the different biological processes, cellular components, and molecular functions associated with the proteins containing differential phosphosites. On the basis of the −log10 *p* value, GO annotation at level three was performed for both the upregulated and downregulated genes. Among the top 20 results, changes in phosphorylation in response to baicalin-mediated interference with rPVL-induced damage in BMECs were analyzed. In the cellular component category, the differentially expressed phosphoproteins (DEPPs) were significantly enriched in the actin cytoskeleton (GO:0015629), tubulin complex (GO:0045298), and microtubule-associated complex (GO:0005875) terms. In the molecular function category, the DEPPs were enriched in actin filament binding (GO:0051015), microtubule plus-end binding (GO:0051010), and Ras GTPase binding (GO:0017016) functions. The identified biological processes related to the experimental phenotypes included the cellular developmental process (GO:0048869), the microtubule-based process (GO:0007017), and the cell cycle process (GO:0022402) (Figure 2C). These findings are consistent with previous reports by our group.

Venn analysis of the dataset revealed significantly changed phosphorylated proteins, namely, P-BCLAF1^S285^, P-CDK7^T170^, P-NF2^S518^, and P-PKM^S37^, which were downregulated after rPVL-induced damage in BMECs but significantly upregulated after baicalin intervention. These results are also shown on a volcano map. Interestingly, these proteins play a significant role in the cell cycle process and regulate cell cycle progression through the modulation of their phosphorylation levels (Figure 2D–F).

### 3.3. Baicalin Alleviated G0/G1 Cycle Arrest in the BMECs Induced by rPVL

**Figure 3:**
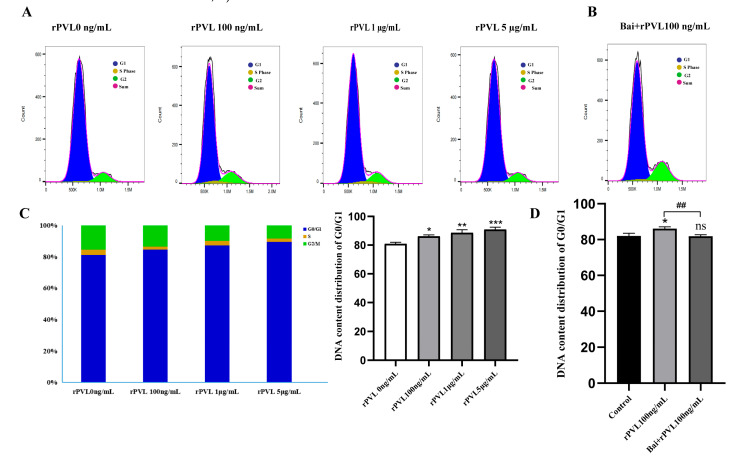
Baicalin alleviated the cell cycle arrest induced by rPVL in BMECs. (**A**) BMECs were treated with increasing concentrations of rPVL for 12 h, and the cell cycle phase was analyzed via flow cytometry. (**B**) The effect of baicalin on the cell cycle phase was assessed through intervention with 5 µg/mL baicalin before 100 ng/mL rPVL treatment in BMECs. (**C**) Distribution of the DNA content in the G1 (blue), S (yellow), and G2 (green) phases of cell cycle progression after rPVL-induced G0/G1 arrest compared with the vehicle control. (**D**) Baicalin alleviated G0/G1 cell cycle arrest in BMECs (* *p* < 0.05, ** *p* < 0.01, *^##^ p* < 0.01, *** *p* < 0.001 and ns *p* > 0.05.

To investigate the impact of rPVL on cell cycle progression, we examined the phase distribution of BMECs treated with varying concentrations of rPVL. To assess whether baicalin can reverse rPVL-induced cell cycle arrest in BMECs, we conducted flow cytometry analysis following baicalin pretreatment. Compared with the control, rPVL caused a dose-dependent increase in the number of BMECs in the G0/G1 phase and a decrease in the number of BMECs in the G2/M phase when the BMECs were treated with 100 ng/mL, 1 μg/mL, or 5 μg/mL rPVL for 12 h (Figure 3A,C). However, intervention with 5 µg/mL baicalin significantly alleviated the G0/G1 phase arrest induced by 100 ng/mL rPVL (Figure 3B,D).

### 3.4. Changes in the Expression of Cell-Cycle-Related Proteins In Vitro

**Figure 4:**
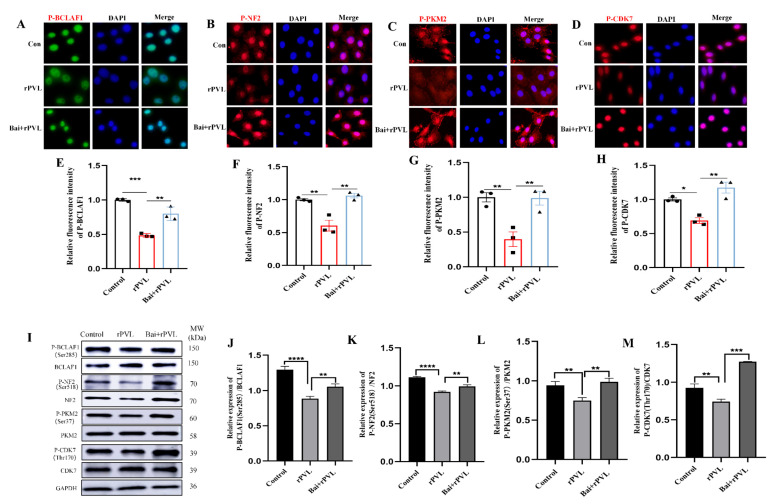
rPVL inhibited the phosphorylation of BCLAF1^S285^, CDK7^T170^, NF2 ^S518^, and PKM2^S37^, whereas baicalin reversed the hypophosphorylation of these cell-cycle-related proteins induced by rPVL. (**A**–**D**) Representative immunofluorescence images of P-BCLAF1^S285^/P-CDK7^T170^/P-NF2^S518^/P-PKM2^S37^. (**E**–**H**) Quantification of immunofluorescence images. The results indicated that after rPVL treatment, the fluorescence intensity of the phosphorylated proteins described above decreased. However, when baicalin was administered prior to rPVL exposure, the inhibitory effect of rPVL on the phosphorylation of these cell-cycle-related proteins decreased, leading to an increase in fluorescence intensity. (**I**) The expression of cell-cycle-related proteins in BMECs was analyzed through Western blotting. (**J**–**M**) Western blot analysis revealed that the phosphorylation levels of BCLAF1^S285^, CDK7^T170^, NF2^S518^, and PKM2^S37^ decreased following rPVL treatment but increased after baicalin intervention. The asterisks (* *p* < 0.05, ** *p* < 0.01, *** *p* < 0.001, and **** *p* < 0.0001) indicate significant differences between groups.

To further confirm that baicalin alleviated rPVL-induced cell cycle arrest by regulating the phosphorylation levels of cell-cycle-related proteins, the expression levels of the phosphorylated proteins BCLAF1^S285^, CDK7^T170^, NF2^S518^, and PKM2^S37^ were assessed through *quantitative* immunofluorescence and Western blot analyses guided by phosphoproteomic profile Venn analysis. To observe the changes in the phosphorylation levels of BCLAF1^S285^, CDK7^T170^, NF2^S518^, and PKM2^S37^ during the cell cycle, immunofluorescence was used to visualize and quantify the phosphorylation levels of these cell-cycle-related proteins before and after baicalin intervention. The results revealed that the fluorescence signal intensity of P-BCLAF1^S285^, P-CDK7^T170^, P-NF2^S518^, and P-PKM2^S37^ in the BMECs decreased after rPVL treatment, whereas the fluorescence intensity of these phosphorylated proteins increased after pretreatment with baicalin (Figure 4A–H). The Western blot analysis of the BMECs treated with rPVL and baicalin supported the immunofluorescence findings presented in Figure 4A–H. In addition, rPVL decreased the level of BCLAF1 phosphorylated at serine 285 (P-BCLAF1^S285^), phosphorylated CDK7 at threonine 170 (P-CDK7^T170^), phosphorylated NF2 at serine 518 (P-NF2^S518^), and phosphorylated PKM2 at serine 37 (P-PKM2^S37^). Furthermore, pretreatment with baicalin led to an increase in the phosphorylation status of these cell cycle regulatory proteins in the presence of rPVL (Figure 4I–M). Collectively, these findings suggested that baicalin alleviated rPVL-induced cell cycle arrest, which was closely related to changes in the phosphorylation levels of cell-cycle-related proteins.

### 3.5. Histopathological Changes in Mammary Tissue and Baicalin-Mediated Regulation of the Dephosphorylation of Cell-Cycle-Regulating Proteins Induced by rPVL In Vivo

**Figure 5:**
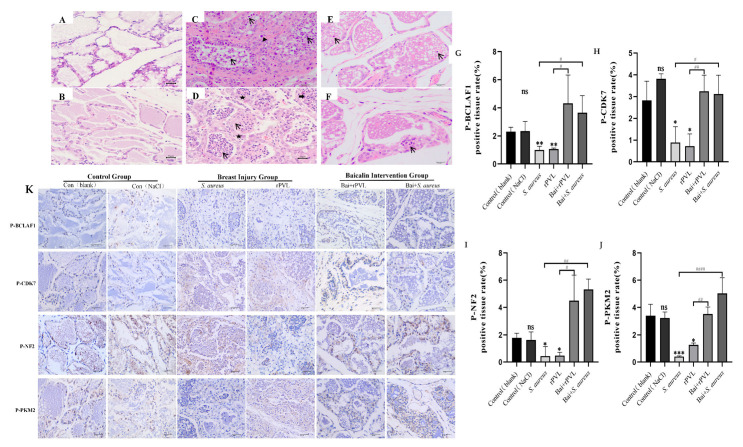
Preemptive intervention with baicalin alleviated the pathological damage to mouse mammary tissue caused by rPVL and reversed the hypophosphorylation of cell cycle regulatory proteins induced by rPVL in the mammary tissue. (**A**–**F**) Histopathology of mammary tissue after infection with *S. aureus* and rPVL (400×). (**A**) Control (blank). (**B**) Control (0.9% NaCl). (**C**) *S. aureus* ATCC49775. (**D**) rPVL. (**E**) Bai+*S. aureus* ATCC49775. (**F**) Bai+rPVL. The baicalin groups were administered 100 mg/kg baicalin. Arrows indicate inflammatory cells, triangles indicate macrophages, thick arrows point to eosinophils, and pentagrams highlight areas of papillary wall edema and thickening. (**K**) Representative immunohistochemical images of BCLAF1^S285^, CDK7^T170^, NF2^S518^, and PKM2^S37^ (400×). (**G**–**J**) The bar charts show the percentages of DAB-positive tissues. The asterisks and well numbers (* *p* < 0.05, ** *p* < 0.01, *^#^ p* < 0.05, *** *p* < 0.001, *^##^ p* < 0.01, ^####^ *p* < 0.0001 and ns *p* > 0.05) indicate significant changes in the expression of the above cell-cycle-regulating proteins in the rPVL-treated group compared with the vehicle group and in the baicalin-treated group compared with the rPVL-treated group, respectively.

Mammary gland tissues were collected at 24 h after treatment and then stained with hematoxylin and eosin. No pathological changes were observed in the control (blank) or control (0.9% NaCl) groups (Figure 5A,B). In the *S. aureus* ATCC49775 group (Figure 5C), numerous inflammatory cells (arrows) and macrophages (triangles) were observed within and around the papillary lumen. Additionally, infiltration of inflammatory cells—including neutrophils and lymphocytes—was noted in the epithelial cells and nodules. In the rPVL group (Figure 5D), the architecture of the mammary tissue was disrupted, with an irregular distribution of mammary papillae. Significant edema and thickening were observed in the papillary walls (pentagrams), accompanied by numerous inflammatory cells (arrows) within the papillary lumen. Scattered eosinophils (thick arrow) were occasionally present. However, after treatment with 100 mg/kg baicalin before *S. aureus* ATCC49775 or rPVL treatment, histopathological impairment was ameliorated, the acinus was not destroyed, and the lobules were complete, although some inflammatory cells were observed locally in the mammary gland tissues (Figure 5E,F).

After a mouse mammary gland injury model was established, the findings of the phosphoproteome were verified at the tissue level via quantitative immunohistochemical analysis, and whether baicalin participated in the effect of the rPVL on the expression of cell-cycle-regulating proteins was investigated. The results demonstrated that rPVL markedly decreased the phosphorylation of BCLAF1^S285^, CDK7^T170^, NF2^S518^, and PKM2^S37^. However, prophylactic treatment with baicalin abolished the dephosphorylation of rPVL (Figure 5B).

## 4. Discussion

Bacterial protein toxins, which are secreted by certain bacteria, cause mild to severe diseases in both humans and animals. Studies have shown that bacterial toxins impair key cellular functions, leading to actin cytoskeleton alterations, the inhibition of cell proliferation, and alterations in the cellular processes that control apoptosis [30]. Milk from cows with mastitis caused by *S. aureus* was reported to have the *pvl* virulence gene, with the highest detection rate reported in recent years, and the detection rate is constantly increasing. A previous study by our research group confirmed that PVL, which is the major toxin of pathogenic *S. aureus* strains, promoted the apoptosis and hyperphosphorylation of key proteins and that baicalin inhibited the apoptosis of BMECs induced by rPVL (recombinant Panton–Valentine leucocidin) [14,15,31]. In this study, high-throughput sequencing, along with comprehensive *in vitro* and *in vivo* experiments, were utilized to identify the key molecules involved and the potential mechanisms through which baicalin prevents rPVL-induced injury to BMECs.

The phosphorylation and dephosphorylation of proteins are key regulatory elements of cellular function that act as molecular switches [32,33]. The phosphoproteome data from this study revealed that the cell cycle pathway remained inactive upon rPVL treatment, as evidenced by the KEGG enrichment results. To investigate whether rPVL’s inhibitory effect on BMEC proliferation involves cell cycle dysregulation, we examined cell cycle phase distributions. The flow cytometry results revealed that the cytotoxic effects of rPVL altered the cellular processes that control the cell cycle and induced G0/G1 phase arrest, whereas early intervention with baicalin promoted the transition of BMECs from the G0/G1 phase to the S and G2/M phases of the cell cycle.

Many cellular events, including protein translation and post-translational modifications, are cell cycle dependent [34]. The differential expression of P-BCLAF1^S285^, P-CDK7^T170^, P-NF2^S518^, and P-PKM2^S37^ between the experimental and control groups was analyzed via bioinformatics. The GO, KEGG, and Venn analyses identified BCLAF1, CDK7, NF2, and PKM2 as the key factors regulating the proliferation of BMECs; however, the changes in the phosphorylation of individual proteins were not statistically significant, but they were borderline enriched, which may be the result of an insufficient sample size used in this study.

Cyclin-dependent protein kinases (CDKs) are crucial regulatory components for the progression of the cell cycle [35]. Cyclin-dependent kinase 7 (Cdk7) is a component of the transcription factor TFIIH and plays a central role in transcription and cell cycle regulation. Cdk7 activates RNA polymerase II through the hyperphosphorylation of its C-terminal domain (CTD). Cdk7 is also a CDK-activated kinase (CAK) that binds to Mat1 and Cyclin H to form active complexes, and it is regulated through phosphorylation at T170, which is located in the activation segment (T loop) [36]. Pyruvate kinase M2 (PKM2) is a protein kinase and a transcriptional coactivator that regulates cell proliferation and migration through post-translational modifications, such as phosphorylation [37]. For example, a mutational SHP2 inhibitor, SHP099, reportedly inhibits tumor proliferation and migration in gastric cancer by dephosphorylating pyruvate kinase M2 (PKM2) protein [38]. The results of the bioinformatics analysis were confirmed via Western blot and immunofluorescence analyses, which revealed that the levels of CDK7 and PKM2 were significantly lower in the rPVL-treated group than in the control group and the baicalin pretreatment group. These results suggested that CDK7 and PKM2 are involved in baicalin-mediated regulation of the cell cycle arrest of BMECs induced by rPVL. Furthermore, the phosphorylation of Ser518 in Merlin expressed by NF2 leads to defects in mitotic spindle localization and delays the transition from metaphase to anaphase in the cell cycle [39]. Protein phosphorylation is an important mechanism for regulating the DNA damage response. BCLAF1 is a key regulator of TNF-α-induced apoptosis [40], and the phosphorylation of BCLAF1 at Ser290 is involved in the regulation of the DNA damage response, eliminating the phosphorylation of BCLAF1 at Ser290 and suppressing gastric cancer (GC) cell proliferation [41]. BCLAF1 has also been reported to mediate cisplatin resistance by regulating the repair of DNA damage and p21-mediated G1 phase arrest in A549/DDP cells [42]. The *in vitro* experiments in this study demonstrated that rPVL decreased the phosphorylation of NF2 and BCLAF1, which are associated with spindle localization and the DNA damage response, whereas baicalin significantly increased their phosphorylation levels. These results were consistent with the results of the GO enrichment analysis of the phosphoproteome. Poor phosphorylation of NF2 and BCLAF1 leads to mitotic abnormalities in BMECs, and the virulence of rPVL delays entry into mitosis for BMECs, whereas abundant phosphorylation of NF2 and BCLAF1, which is regulated by baicalin, promotes entry into mitosis for these cells. These results collectively suggest that baicalin may regulate the cell cycle and alleviate rPVL-induced proliferation inhibition in BMECs, indicating its potential role in preventing early-stage bovine mastitis.

To further assess the actual effects of rPVL on the mammary gland and the pre-protective function of baicalin *in vivo*, animal experiments were conducted. The *in vivo* experiments confirmed that baicalin exhibited a protective effect against mammary tissue damage in the mice induced by *S. aureus* ATCC49775 and rPVL, enhancing the structural integrity of the mammary gland while reducing inflammatory cell infiltration. Furthermore, quantitative immunohistochemical analyses revealed significant decreases in the phosphorylation of BCLAF1, CDK7, NF2, and PKM2 following rPVL treatment. In contrast, marked increases in the phosphorylation of these critical cell cycle regulatory proteins were observed after baicalin intervention.

Although this study elucidates the protective role of baicalin in alleviating rPVL-induced cell cycle arrest and mitigating mammary gland injury, several limitations remain. These limitations include the limited sample size for omics analyses and the potential off-target effects of baicalin. Furthermore, the mechanism through which baicalin protects against PVL-induced BMEC damage—specifically, whether it involves the inhibition of LukS-PV attachment—requires further investigation.

## 5. Conclusions

In conclusion, this investigation of the phosphoproteome via mass spectrometry revealed that baicalin can protect against rPVL-mediated injury in BMECs and is closely associated with the regulation of protein phosphorylation levels. The *in vivo* experiments in this study indicated that baicalin significantly attenuates pathological damage in the mammary glands induced by *S. aureus* ATCC49775 and rPVL, demonstrating a protective effect on mammary gland tissues during *S. aureus-* and rPVL-induced mastitis. Flow cytometry analysis demonstrated that rPVL inhibits BMEC proliferation through G0/G1 phase arrest. Furthermore, comprehensive *in vitro* and *in vivo* experiments demonstrated that the alleviating effect of baicalin on the cell cycle arrest induced by rPVL in BMECs is closely related to its regulatory effects on the phosphorylation levels of cyclins P-CDK7^T170^, P-BCLAF1^S285^, P-NF2^S518^, and P-PKM2^S37^. These data provide a basis for multidisciplinary research, including investigations into the mechanisms through which baicalin counteracts bacterial toxins and its potential development as a livestock feed additive. In addition to its promising application for preventing bovine mastitis, the potential intervention value of baicalin against other pore-forming toxin (PFT)-related infections warrants further investigation.

## Data Availability

The original contributions presented in this study are included in the article/Supplementary Material. Further inquiries can be directed to the corresponding authors.

## Supplementary Materials

The following supporting information can be downloaded at: https://www.mdpi.com/article/10.3390/microorganisms13071673/s1, Figure S1: Vector Information of LukF-PV; Figure S2: Restriction analysis of recombinant plasmids of LukF-PV (Digested with NdeI-XhoI); Figure S3: SDS-PAGE analysis of pilot-scale fusion protein expression of LukF-PV; Figure S4: SDS-PAGE analysis of LukF-PV-EGFP fusion protein purified by Nickel-Agarose Affinity Chromatography; Figure S5: SDS-PAGE Analysis of the Final Purified LukF-PV; Figure S6: Western Blot Analysis of the Final Purified Protein LukF-PV; Figure S7: Vector Information of LukS-PV; Figure S8: Restriction analysis of recombinant plasmids of LukS-PV (Digested with NdeI-XhoI); Figure S9: SDS-PAGE analysis of pilot-scale fusion protein expression of LukS-PV; Figure S10: SDS-PAGE analysis of LukS-PV-EGFP fusion protein purified by Nickel-Agarose Affinity Chromatography; Figure S11: SDS-PAGE Analysis of the Final Purified LukS-PV; Figure S12: Western Blot Analysis of the Final Purified Protein LukS-PV.
