# Supplementary material for: Phosphoproteome Reveals the Role of Baicalin in Alleviating rPVL-Induced Cell Cycle Arrest in BMECs
Source: Microorganisms. 2025 Jul 16;13(7):1673. doi: 10.3390/microorganisms13071673 (PMC12300862; doi:10.3390/microorganisms13071673)
Supplement: Supplementary file 1 [file microorganisms-13-01673-s001.zip › Supplementary materials S1.pdf]

Prokaryotic Expression and Purification Process of LukF-PVL

1. Sequence information

1.1 gene sequence

GCTCAACATATCACCCCTGTCAGTGAAAAGAAGGTTGATGATAAAATTACACTTTATAAGACGACTGCTACGTCGGACTCG

GATAAGCTGAAGATTTCACAGATCCTGACCTTTAATTTTATCAAAGACAAAAGCTATGACAAGGATACTTTGATCTTAAAGGCAG

CCGGAAATATCTATAGCGGCTACACCAAACCAAACCCCAAGGACACGATTAGCAGCCAGTTTTATTGGGGTTCCAAGTACAACAT

TTCAATCAATAGTGACTCGAATGATTCTGTTAACGTTGTTGATTATGCACCGAAGAATCAGAACGAAGAGTTTCAGGTTCAGCAG

ACTGTAGGTTATAGCTACGGAGGTGACATTAACATTTCAAACGGATTGTCCGGCGGGGGAAACGGATCTAAATCCTTTTCCGAA

ACGATTAACTACAAACAGGAGTCCTATCGTACGTCCTTGGATAAACGTACAAATTTCAAAAAGATTGGTTGGGATGTCGAAGCTC

ATAAAATTATGAATAATGGGTGGGGCCCTTATGGCCGCGACAGTTATCACAGCACCTATGGAAACGAAATGTTCTTGGGTTCACG

CCAGTCGAACCTGAATGCTGGGCAGAACTTTCTGGAATACCACAAAATGCCTGTACTTAGTCGCGGCAACTTTAATCCTGAGTTT

ATTGGCGTGCTGAGTCGCAAGCAGAATGCGGCTAAGAAGAGCAAGATCACCGTGACTTACCAACGCGAAATGGACCGTTACAC

CAATTTCTGGATTAATTTCAACTGGATTGGGAATAATTACAAAGATGAAAACCGCGCTACCCACACTTCAATCTACGAAGTTGATT

GGGAAAATCACACCGTAAAACTTATCGACACACAGAGCAAAGAGAAAAACCCAATGAGTTAA

1.2 Vector information

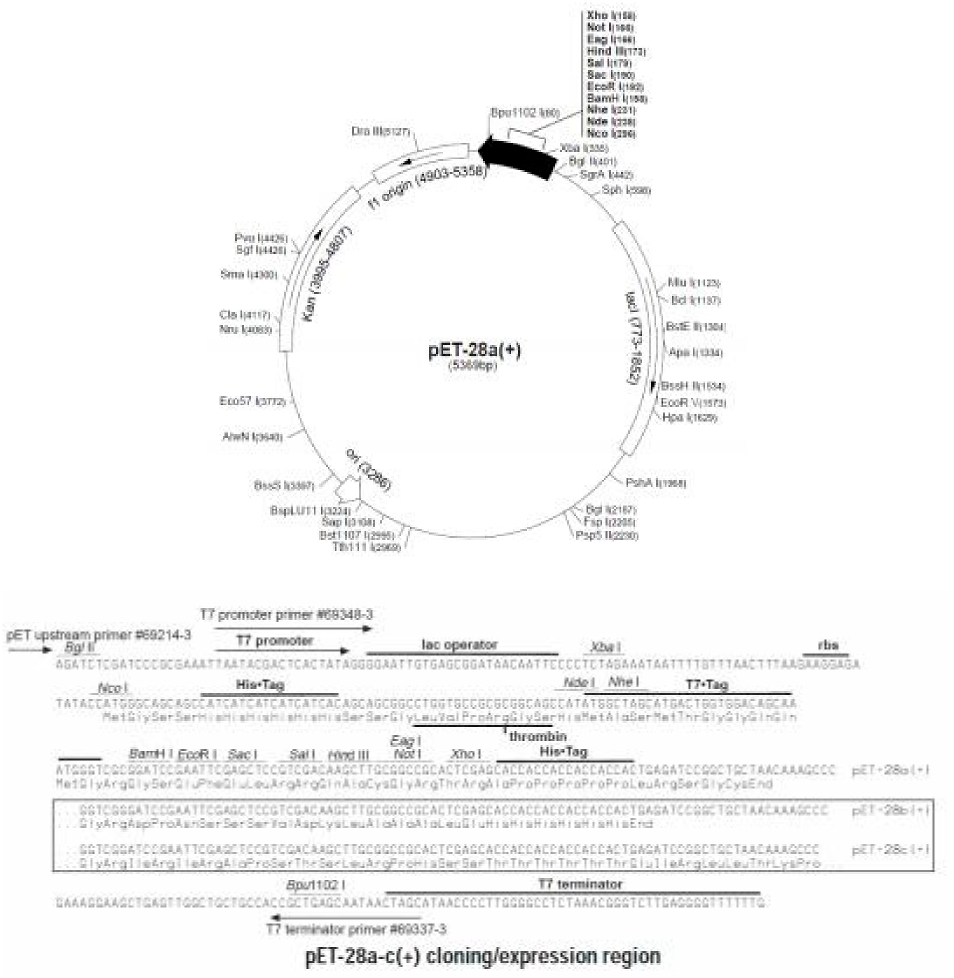

Figure S1. Vector Information of LukF-PV

1.3 Expressed amino acid sequence

Protein Length=322 MW=36733.1

Predicted pI=9.49

MGSSHHHHHHSSGLVPRGSHM

AQHITPVSEKKVDDKITLYKTTATSDSDKLKISQILTFNFIKDKSYDKDTLILKAAGNIYSGYTKPNPKDTISSQFYWGSKYNISINSDSND

SVNVVDYAPKNQNEEFQVQQTVGYSYGGDINISNGLSGGGNGSKSFSETINYKQESYRTSLDKRTNFKKIGWDVEAHKIMNNGWG

PYGRDSYHSTYGNEMFLGSRQSNLNAGQNFLEYHKMPVLSRGNFNPEFIGVLSRKQNAAKKSKITVTYQREMDRYTNFWINFNWI

GNNYKDENRATHTSIYEVDWENHTVKLIDTQSKEKNPMS

2.Method

The LukF-PVL protein gene sequence of PV-leukocidin was inserted into the pET28a expression vector, and the vector was transformed into *E. coli* receptor cells, which were heat-excited at 42 °C. The cells were streaked onto agar plates containing 30 μg/mL kanamycin and cultured at 37 °C, and induced to express. The recombinant LukF-PVL protein was subjected to affinity purification and the purity was confirmed using SDS-PAGE and western blotting (purity > 90%).

3.Results

3.1 Restriction analysis of recombinant plasmid

Marker：SM0331，Thermo

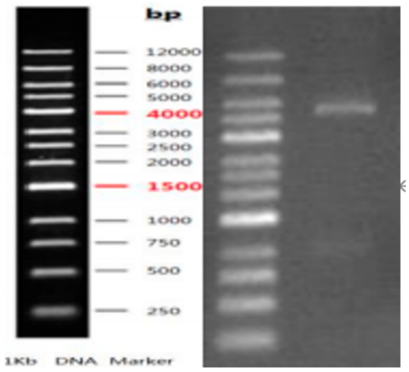

Figure S2. Restriction analysis of recombinant plasmids of LukF-PV (Digested with NdeI-XhoI)

3.2 Detection of LukF-PV protein expression

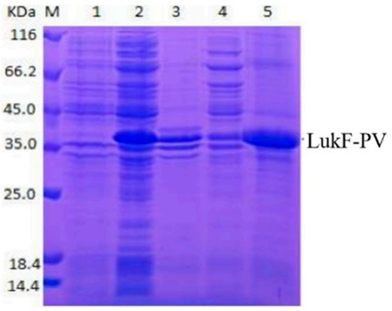

Figure S3. SDS-PAGE analysis of pilot-scale fusion protein expression of LukF-PV

M:Protein Marker;1.Pre-induction total lysate; 2.20°C supernatant; 3. 20°C pellet; 4. 37°C supernatant; 5. 37°C pellet

3.3 Protein purification analysis

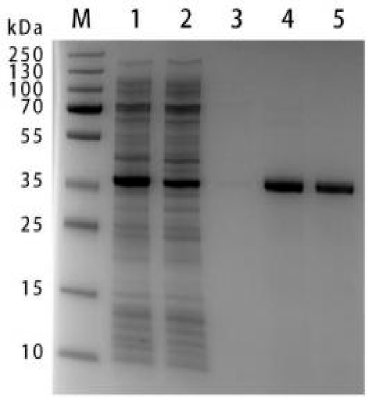

Figure S4. SDS-PAGE analysis of LukF-PV-EGFP fusion protein purified by Nickel-Agarose Affinity Chromatography

M：Protein marker；1: Load; 2: Flow-through; 3: 20 mM Imidazole elution; 4: 250 mM Imidazole elution; 5: 500 mM Imidazole elution

3.4 Verification of Target Fusion Protein

The purified fusion protein displayed a distinct band near the theoretical molecular weight position on SDS-PAGE analysis, providing preliminary confirmation of successful purification.

To further confirm that the purified protein is the target protein, it was stained using the TMB color development kit and analyzed according to the Western blot procedure. The results revealed a distinct band at the expected position, indicating that this protein is the target protein LukF-PV.

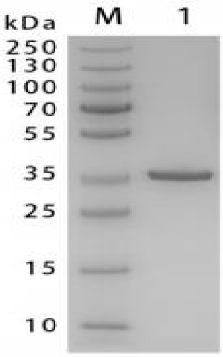

Figure S5. SDS-PAGE Analysis of the Final Purified LukF-PV

M:Protein marker；1. LukF-PV

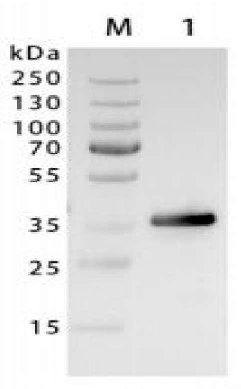

Figure S6． Western Blot Analysis of the Final Purified Protein LukF-PV

M: Protein marker； 1. LukF-PV
