# Supplementary material for: Phosphoproteome Reveals the Role of Baicalin in Alleviating rPVL-Induced Cell Cycle Arrest in BMECs
Source: Microorganisms. 2025 Jul 16;13(7):1673. doi: 10.3390/microorganisms13071673 (PMC12300862; doi:10.3390/microorganisms13071673)
Supplement: Supplementary file 1 [file microorganisms-13-01673-s001.zip › Supplementary materials S2.pdf]

SProkaryotic Expression and Purification Process of LukS-PVL

1. Sequence information

1.1 gene sequence

TTTCACGAGAGCAAAGCAGACAATAACATTGAGAATATCGGGGATGGCGCAGAGGTCGTAAAACGTACTGAAGACACTTC

ATCCGACAAGTGGGGCGTCACGCAAAACATCCAATTCGATTTTGTTAAAGATAAGAAGTATAACAAGGACGCATTGATCTTAAAA

ATGCAGGGCTTTATCAACAGCAAGACTACATACTACAACTACAAGAATACTGATCACATTAAAGCCATGCGTTGGCCTTTTCAGTA

TAACATCGGTTTAAAAACGAACGACCCTAATGTAGATTTGATCAACTATTTGCCGAAAAATAAAATCGATTCGGTAAACGTGTCCC

AGACTTTAGGGTACAATATCGGGGGCAATTTTAATTCAGGCCCTTCAACAGGCGGTAATGGAAGTTTTAACTACTCAAAAACCAT

CTCGTATAATCAGCAGAACTACATTTCCGAGGTAGAACATCAGAACAGCAAGTCAGTGCAGTGGGGTATCAAGGCCAACTCTTT

CATTACCTCGTTGGGAAAAATGTCGGGTCATGATCCCAATCTGTTCGTCGGATACAAACCATATAGCCAGAATCCCCGTGACTACT

TCGTCCCCGATAATGAATTACCCCCGTTAGTTCATAGCGGGTTCAACCCTTCGTTCATCGCAACCGTTTCACACGAAAAAGGCTCT

GGTGACACAAGTGAATTTGAGATCACATATGGACGTAATATGGATGTTACCCATGCGACGCGCCGTACTACACACTATGGTAACA

GTTATTTAGAGGGCTCGCGTATCCACAATGCGTTTGTAAATCGTAATTACACCGTCAAGTATGAAGTAAATTGGAAGACGCACGA

GATCAAGGTAAAAGGTCACAATTAA

1.2 Vector information

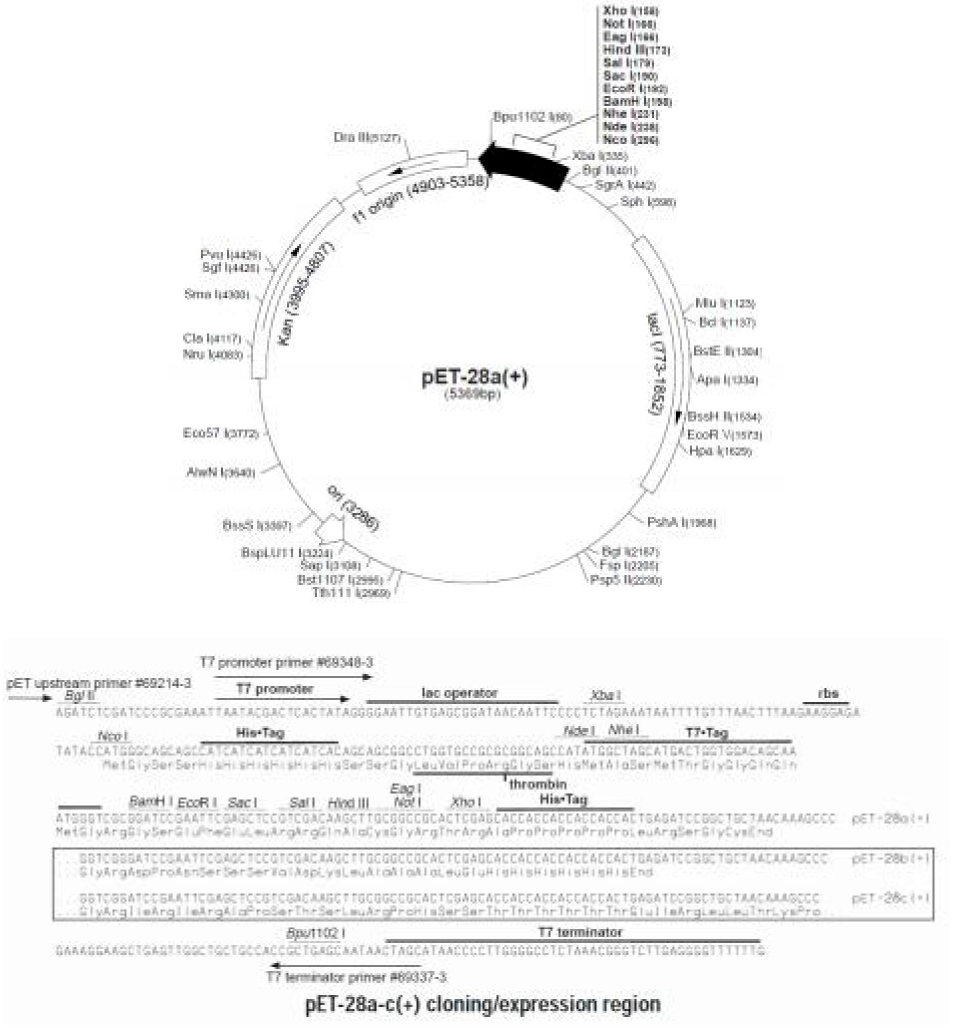

Figure S7. Vector Information of LukS-PV

1.3 Expressed amino acid sequence

Protein Length=311 MW=35302.6

Predicted pI=9.30

MGSSHHHHHHSSGLVPRGSHM

FHESKADNNIENIGDGAEVVKRTEDTSSDKWGVTQNIQFDFVKDKKYNKDALILKMQGFINSKTTYYNYKNTDHIKAMRWPFQYN

IGLKTNDPNVDLINYLPKNKIDSVNVSQTLGYNIGGNFNSGPSTGGNGSFNYSKTISYNQQNYISEVEHQNSKSVQWGIKANSFITSL

GKMSGHDPNLFVGYKPYSQNPRDYFVPDNELPPLVHSGFNPSFIATVSHEKGSGDTSEFEITYGRNMDVTHATRRTTHYGNSYLEG

SRIHNAFVNRNYTVKYEVNWKTHEIKVKGHN

2.Method

The LukS-PVL protein gene sequence of PV-leukocidin was inserted into the pET28a expression vector, and the vector was transformed into *E. coli* receptor cells, which were heat-excited at 42 °C. The cells were streaked onto agar plates containing 30 μg/mL kanamycin and cultured at 37 °C, and induced to express. The recombinant LukS-PVL protein was subjected to affinity purification and the purity was confirmed using SDS-PAGE and western blotting (purity > 90%).

3.Results

3.1 Restriction analysis of recombinant plasmid

Marker：SM0331，Thermo

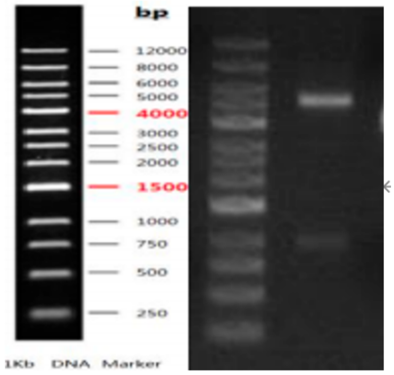

Figure S8. Restriction analysis of recombinant plasmids of LukS-PV (Digested with NdeI-XhoI)

3.2 Detection of LukS-PV protein expression

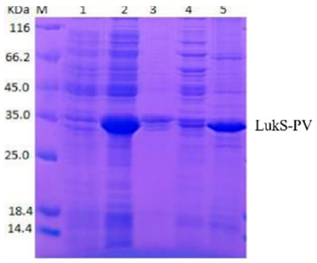

Figure S9. SDS-PAGE analysis of pilot-scale fusion protein expression of LukS-PV

M:Protein Marker;1.Pre-induction total lysate; 2.20°C supernatant; 3. 20°C pellet; 4. 37°C supernatant; 5. 37°C pellet

3.3 Protein purification analysis

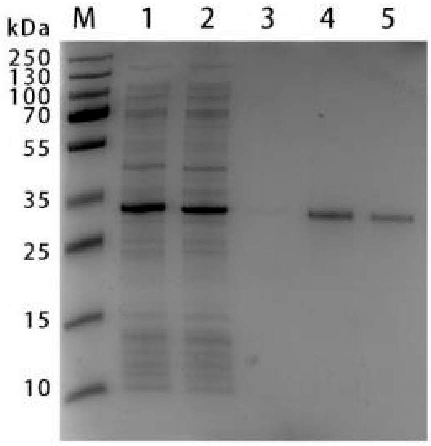

Figure S10. SDS-PAGE analysis of LukS-PV-EGFP fusion protein purified by Nickel-Agarose Affinity Chromatography

M：Protein marker；1: Load; 2: Flow-through; 3: 20 mM Imidazole elution; 4: 250 mM Imidazole elution; 5: 500 mM Imidazole elution

3.4 Verification of Target Fusion Protein

The purified fusion protein displayed a distinct band near the theoretical molecular weight position on SDS-PAGE analysis, providing preliminary confirmation of successful purification.

To further confirm that the purified protein is the target protein, it was stained using the TMB color development kit and analyzed according to the Western blot procedure. The results revealed a distinct band at the expected position, indicating that this protein is the target protein LukS-PV.

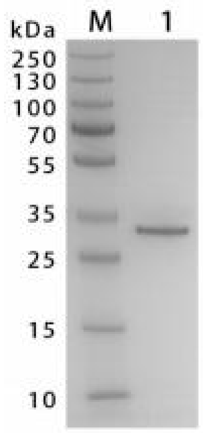

Figure S11. SDS-PAGE Analysis of the Final Purified LukS-PV

M：Protein marker；1. LukS-PV

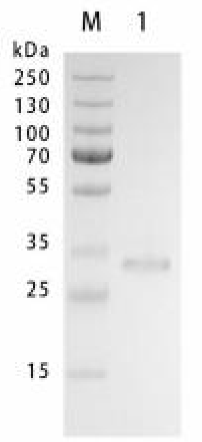

Figure S12．Western Blot Analysis of the Final Purified Protein LukS-PV

M：Protein marker；1. LukS-PV
